# Pinpointing transcription factor binding sites from ChIP-seq data with SeqSite

**DOI:** 10.1186/1752-0509-5-S2-S3

**Published:** 2011-12-14

**Authors:** Xi Wang, Xuegong Zhang

**Affiliations:** 1 MOE Key Laboratory of Bioinformatics and Bioinformatics Division, TNLIST / Department of Automation, Tsinghua University, Beijing 100084, China; 2 Current address: School of Biomedical Sciences and Pharmacy, The University of Newcastle, University Drive, Callaghan NSW 2308, Australia

## Abstract

**Background:**

Chromatin immunoprecipitation combined with the next-generation DNA sequencing technologies (ChIP-seq) becomes a key approach for detecting genome-wide sets of genomic sites bound by proteins, such as transcription factors (TFs). Several methods and open-source tools have been developed to analyze ChIP-seq data. However, most of them are designed for detecting TF binding regions instead of accurately locating transcription factor binding sites (TFBSs). It is still challenging to pinpoint TFBSs directly from ChIP-seq data, especially in regions with closely spaced binding events.

**Results:**

With the aim to pinpoint TFBSs at a high resolution, we propose a novel method named SeqSite, implementing a two-step strategy: detecting tag-enriched regions first and pinpointing binding sites in the detected regions. The second step is done by modeling the tag density profile, locating TFBSs on each strand with a least-squares model fitting strategy, and merging the detections from the two strands. Experiments on simulation data show that SeqSite can locate most of the binding sites more than 40-bp from each other. Applications on three human TF ChIP-seq datasets demonstrate the advantage of SeqSite for its higher resolution in pinpointing binding sites compared with existing methods.

**Conclusions:**

We have developed a computational tool named SeqSite, which can pinpoint both closely spaced and isolated binding sites, and consequently improves the resolution of TFBS detection from ChIP-seq data.

## Introduction

Exploring protein-DNA binding events in a genome-wide manner is a key step in studying transcription regulation. Chromatin immunoprecipitation (ChIP) [1] followed by hybridization to DNA tiling arrays (ChIP-chip) [2-4] or by next-generation high-throughput sequencing (ChIP-seq) [5-9] are major techniques for experimentally profiling the binding events. Due to many advantages of next-generation sequencing [10,11], ChIP-seq measures immunoprecipitated DNA fragments at a higher signal-to-noise ratio than ChIP-chip, and provides the potential to detect protein-DNA binding locations at a higher resolution [9]. ChIP-seq is being widely used on large mammalian genomes to comprehensively map *in vivo* transcription factor (TF)-binding events [6,12] and histone marks [5,13].

In ChIP-seq, DNA molecules are isolated after protein-DNA cross-linking, and randomly sheared into fragments by sonication, followed by immunoprecipitation. After cross-link reversal, immunoprecipitated (IPed) DNA fragments are sequenced with next-generation sequencing machines, such as the Illumina Genome Analyzer. Details of the experimental procedures can be found in recent literature such as [14,15]. Supplementary Figure S1 in Additional File 1 shows a typical workflow of ChIP-seq experiment. The experiment produces large amounts of short reads measured from 5’end of each IPed double-strand DNA fragment. Typical read lengths are 25-36 nucleotides (nt) as in profiling of transcription factors binding events on the human genome by the ENCODE project [16,17]. The sequenced reads are then mapped to the reference genome. Unmapped reads are excluded in following analyses, and those reads that can be mapped to multiple locations are also filtered out in most applications. The remaining uniquely mapped reads (called tags) assigned with genomic positions and orientations that provide the information of the starting sites and directions of the IPed DNA fragments.

Transcription factors recognize and bind to specific DNA sequences (named motifs, typically about 5~20 base-pair (bp) long) [18,19]. The binding locations are called transcription factor binding sites (TFBSs). A major purpose of ChIP-seq experiments with transcription factors is to accurately identify TFBSs on the genome. Several computational methods and tools have been developed to detect TFBSs from ChIP-seq data. The most straight-forward method is to count tags along the genome and detect TFBSs or binding regions that cover them with highly accumulated reads (e.g., [6]). However, as ChIP-ed DNA fragments are of ~150-300-bp long but the sequencing reads are much shorter, the location detected in this way will be very imprecise. A better strategy is to extend each tag to a certain length (usually the estimated fragment length) along its strand orientation to generate “pseudo fragments” (composing the “extended set” or “XSET” [20]). Such pseudo fragments are then profiled along the genomic coordinates, and TFBSs can be detected from profile peaks. Methods taking this type of strategy include PeakSeq [21], FindPeaks [22], F-Seq [23], GLITR [24] and HPeak [25]. The extension step works similar to a low-pass filtering in signal processing, which therefore results in a low resolution in detection of peaks. More sophistical methods were developed to improve the resolution of TFBS detection without extending tags [26]. For example, CisGenome [27], QuEST [28], MACS [29] and USeq [30] take similar strategies to shift both forward and reverse tag profiles toward the center to form an integrated tag profile for peak calling; SISSRs [31] calculates read counts of the forward and reverse strands as positive and negative signals, respectively, and takes the transition point as the candidate site; spp [32] takes into account the symmetry of the two-stand tag profiles and detects paired peaks. Pepke et al [26] gives a comprehensive review on these methods and discussed the need of peak deconvolution when there may be multiple binding sites in one peak region. Laajala *et al*. [33] and Wilbanks & Facciotti [34] conducted series of comparative experiments with most of the open-source methods. They observed no substantial difference between the compared methods in the sensitivity and specificity of binding region detection, but noticeable divergence was observed in their spatial resolutions for precisely locating binding sites.

In many cases, TFs can bind to a genomic region at closely spaced adjacent sites. Pinpointing TFBSs from ChIP-seq data is still quite challenging in such cases. For example, taking the case of two adjacent binding sites as an example, many existing methods tend to identify a single site around the two binding sites’ center, or report one region covering both sites [26], leaving binding site detection for a secondary step of sequence motif analysis. Due to the possible indirect binding of some TFs and the influence of other factors such as the chromatin structure, there are cases that TFBS sequence motifs cannot be found in the detected region and also cases when sequence motifs do not imply real binding events. There is a need to develop methods that can locate multiple adjacent TFBSs from ChIP-seq data at a higher resolution [26]. In this paper, we developed a method called SeqSite to pinpoint TFBSs for both isolated and close adjacent binding events from ChIP-seq data. The method includes a second step for locating binding sites by modeling the tag density profile after binding regions are detected in the first step. A series of simulation experiments were conducted to study the property and performance of the method. The method SeqSite was applied on three published datasets of transcription factors GABP [28], STAT1 [21] and NRSF [28]. Experiments show that it performs better than or similarly with existing methods on the accuracy of binding site detection, but SeqSite can locate binding sites at a higher resolution and can pinpoint adjacent binding sites even if they are as close as 40-bp from each other. Recent publications reported three new methods, CSDeconv [35], PICS [36] and GPS [37], for similar questions. We compared them and some other classical tools with SeqSite in our experiments and discussed the characteristics and advantages of our method. The software tool SeqSite implemented in C/C++ code is freely available at SeqSite website [38].

## Results

### Characteristics of ChIP-seq data for multiple adjacent TFBSs

**Figure 1:**
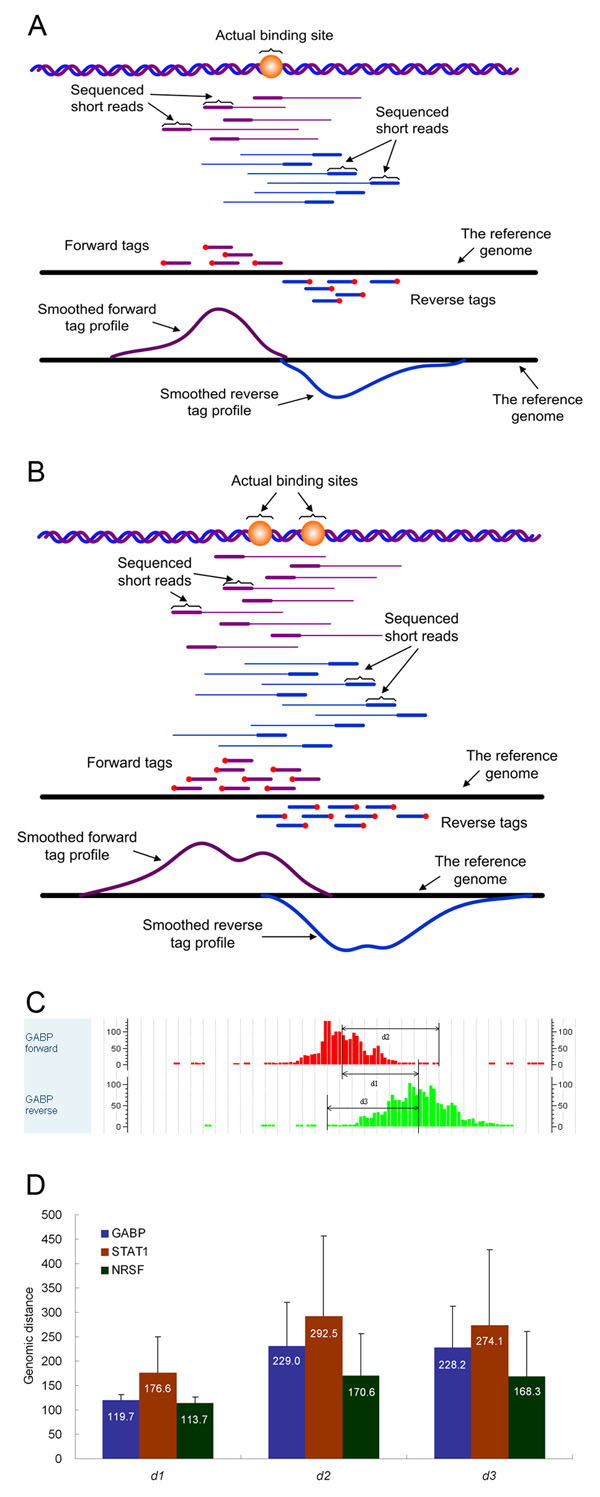
**The evidence for clustered adjacent binding sites.** (A) The sketch illustrates the forward and reverse tag pileups and smoothed tag profiles resulting from an isolated binding site. (B) The sketch shows the forward and reverse tag pileups and smoothed tag profiles resulting from two nearby binding sites in a binding region. It can be seen that the overlapping of the forward and reverse tag pileups is mainly due to adjacent binding sites in the binding region. (C) A snapshot from CisGenome Browser showing an example of the overlapped forward and reverse tag pileups. The example is from the genomic regions of chr22:29885800-29886400 of the GABP ChIP-seq data. Also illustrated are the three distances (*d*_1_, *d*_2_ and *d*_3_) defined in the main text. (D) The average values and standard deviations of *d*_1_, *d*_2_ and *d*_3_ based on top 5% dense tag-enriched regions of GABP, STAT1 and NRSF ChIP-seq data, respectively.

In the ChIP-seq protocol with Illumina Genome Analyzer [14,15], the ChIP-ed DNA fragments are sequenced from either end randomly. So, mappable short reads can be mapped to the reference genome on the forward or the reverse strand with equal probability. Figure 1A shows a typical tag distribution resulting from a single binding event. Since ChIP-ed DNA fragments always cover binding sites, the forward tags are not expected to start from downstream locations of a binding site, and (the right-most positions of) the reverse tags are not expected to start from the upstream regions. Besides, DNA fragmentation is operated after protein-DNA cross-linking, so these cross-linked regions cannot be sheared. As a result, in ideal cases, sequencing tags on the forward strand should all start from upstream locations of a binding site, and those on the other strand should all start from downstream locations in a symmetric manner. For an isolated binding site, the region containing forward tags should not be overlapped with the region piled-up with reverse tags (Figure 1A). However, this is not always the case observed on many real ChIP-seq datasets. For example, we observed many overlapping regions from the GABP ChIP-seq dataset (see Methods for descriptions of the dataset). Figure 1C shows an example. Besides the effect of possible noises, the major reason for this phenomenon is the existence of multiple binding sites close to each other in a short region. Figure 1B illustrates how two adjacent binding sites form the overlapped tag signal.

We further demonstrated the ubiquitous phenomenon of closely spaced binding sites by investigating ChIP-seq datasets of transcription factors GABP, STAT1 and NRSF (see Methods). We used a tag-clustering method to get pairs of neighboring tag clusters on the specific strands, and defined *tag density* as a measure of the signal intensity to rank all tag clusters (see Methods for details). We focused on genomic regions ranked high to ensure that they are truly binding regions. We defined three variables to characterize the overlapping situation of tag stacks on the two strands. As shown in Figure 1C, variable *d*_1_ measures the genomic distance between the density centers of two tag stacks on opposite strands. Variable *d*_2_ measures the lag after the center on the forward strand (the distance from the center to the ending of the tag stack along the sequencing direction) and *d*_3_ measures the lag on the reverse strand. We estimated the boundary of a tag stack by comparing with the background signal (see Methods for details). Figure 1D shows average *d*_1_, *d*_2_ and *d*_3_ with standard deviations in the top 5% densest tag clusters in each dataset, and Supplementary Figure S2 in Additional File 1 provides the results based on other parameter settings. We can see that the averages of *d*_2_ and *d*_3_ in all three datasets are larger than the averages of *d*_1_, which indicates that many TF-binding regions must contain multiple adjacent binding sites.

The observed phenomenon is consistent with previous findings that around half of the binding regions identified by existing methods actually contain two or more associated binding motifs [28,31].

### The SeqSite method

We developed a method called SeqSite for detecting binding regions and pinpointing closely spaced adjacent binding sites in detected binding regions. It first aggregates tags to form tag clusters on each strand. Then the Poisson models with dynamic parameters are adopted to assess the statistical significance of tag enrichment comparing to the control data or local background. SeqSite filters out tag clusters that are not significant according to a given false discovery rate. Tag clusters with fewer than 10 tags or shorter than 100-nt are also excluded, for those tag cluster regions are probably artifacts [26] and too noisy for the secondary binding site detection. The remaining tag clusters are reported as TF binding regions with refined boundaries. This is the first step of the method.

**Figure 2:**
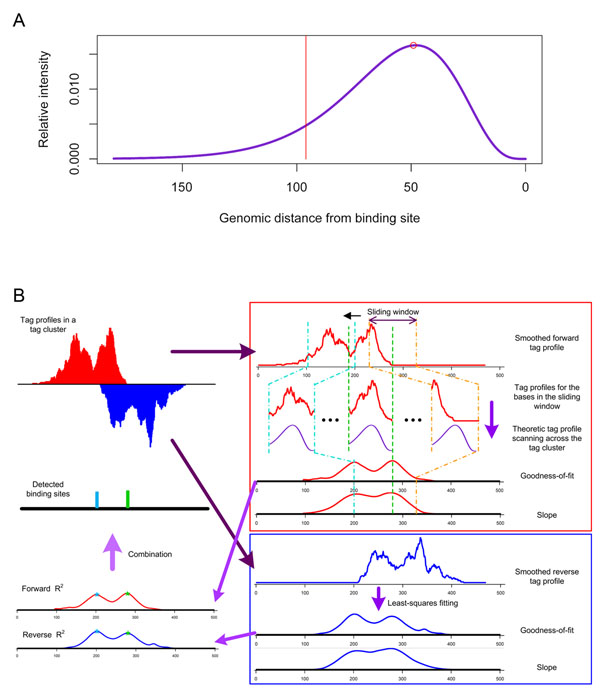
**Modeling tag density profile and the detection of binding sites.** (A) Shown is the tag density profile resulting from a single binding site. The binding site is at position 0. In this example, the average DNA fragment length is 120 bp, and the peak of this curve is at the position 49 bp from the assumed binding site. We use only part of the tag density curve (the right side of the red line) when detecting binding sites. (B) Shown is the schema of binding site detection. Given the modeled tag density profile, a least-squares fitting strategy is adopted to detect binding sites for both the forward and reverse strands. We take the goodness of fit as the criterion to identify binding sites, and the associated regression slope as the relative binding affinity. The two DNA strands are processed separately and the results from the both strand are then combined.

The second step of SeqSite detection is to pinpoint binding sites in putative binding regions. SeqSite first estimates the average length *L* of ChIP-ed DNA fragments in the dataset using a gamma distribution and models the tag density profile of a binding site. In a given tag sets, we use the starting point of each tag (5’-end of each read) to represent the tag, avoiding the effect of unwanted low-pass filtering of sequence reads, and get tag profiles in binding regions. A least-squares model-fitting strategy is then applied on each binding region to detect the most likely binding sites. The profile is fitted with the tag signal in a scanning window of width *W*. *W* is set to be close to but smaller than *L* to take into account the effect of fragment size selection in ChIP-seq protocol [14,15]. A goodness-of-fit is calculated at each genomic location when the window is sliding in the region, resulting in a goodness-of-fit curve. Strong peaks on the curve indicate possible binding sites, and the slope in the fitting associated with the most likely site is use to indicate the relative binding affinity of the site. The detection procedure is applied on both strands and results are merged by some rules. Figure 2 illustrates the principle of the SeqSite method and demonstrates the detection of multiple adjacent binding sites in a binding region. Details of the method are described in the Methods section.

Supplementary Figure S3 in Additional File 1 gives the diagram of the whole system. We implemented SeqSite in C/C++ code with efficient algorithm design. The software tool SeqSite can be downloaded for free academic use at SeqSite website [38].

### Binding site detection on simulated data

**Figure 3:**
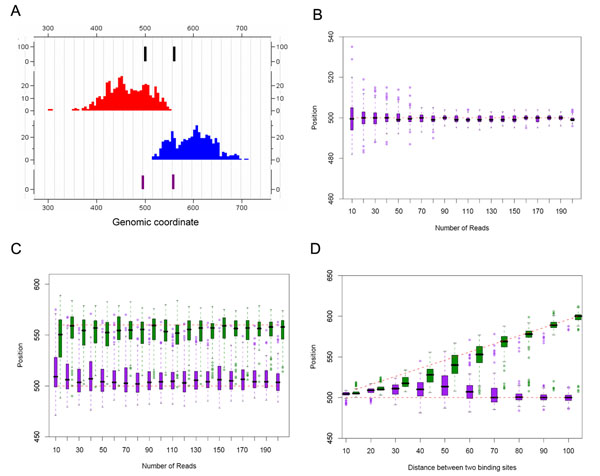
**Simulation results.** (A) An example of the simulated data. The first track shows the assumed binding sites; the following two tracks are the forward and the reverse tag profiles, respectively; and the last track is the binding sites identified by SeqSite. In this case, the assumed binding site coordinates were 500 and 560, and the detected binding sites were at 495 and 558. (B-D) Shown are box-plots of the positions of detected binding sites in different simulated cases. In each panel, the vertical axis represents the genomic position, with red dashed lines showing the true binding locations in the simulation models. Each box-plot shows the results from 100 repeats. (B) Results for single binding site situations. The horizontal axis represents the sequencing depth represented by the number of tags on each strand in the simulated data. (C) Results for two binging sites of distance 60 bp, with different sequencing levels. (D) Results for two binging sites with fixed read count 100. The horizontal axis represents the distance between the two binding sites in the simulation model.

We conducted a series of simulation experiments to study the property of the proposed method, as true answers are not available for real ChIP-seq data. Here we focused on the locating of multiple binding sites within a binding region, i.e., the major purpose is to see how well SeqSite performs in pinpointing binding sites given binding regions. Different sequencing depths and the distance between two adjacent binding sites were simulated. We set the average DNA fragment size to be 120-bp in the simulation, according to the estimated values in the GABP and NRSF datasets (Table 1). Details of the simulation procedures are described in Methods. Figure 3A shows an example of the simulated data that contains two binding sites in a binding region. Each experiment with the same setting was repeated 100 times to estimate the variance of the results.

**Table 1:** Summary of the SeqSite results on the three ChIP-seq datasets at FDR 10%.

**TF**	***L***	**# BRs**	**# BSs**	**# BRs with multi-BS (%)**
GABP	114.6	12,875	28,846	9,786 (76.0)
STAT1	194.6	39,222	82,104	23,914 (61.0)
NRSF	107.0	3,786	6,047	1,948 (51.5)

*L*: average DNA fragment size (bp) estimated; BR: binding region; BS: binding site.

We first studied the effect of sequencing depth on the resolution of detecting the binding site locations. We generated 10 to 200 tags for each binding region on one strand, which covers most (82.8%) of the situations in the real data (Supplementary Figure S4 in Additional File 1 shows the distribution on the real data). Figure 3B shows the distribution of the detected binding sites at different sequencing depths for a single binding site. We can see that the detected binding sites center at the true binding site location. Increasing the sequencing depth can improve the resolution but SeqSite can work reasonably well even when the coverage is low. When there are more than 70 tags in the binding region on one strand, most (>97%) of detected binding sites are within 5 bp from the true binding site. When the coverage drops to only 10 tags on each strand, the variance in the estimated site locations becomes larger, but still in 78% of the experiments, the distance between the detected position and the true binding site is less than 10 bp. For cases when there are two adjacent binding sites apart by 60 bp as the example shown in Figure 3C, we observed that the SeqSite successfully distinguish two binding sites except for the lowest sequencing depth, although the locations tend to be closer to each other than the true sites. The effect of sequencing depth is minor unless it is too low. Except for the cases with only 10 tags in the region, more than 70% of the detected sites are within 20 bp from the true binding sites.

We then fixed the number of tags in each binding region as 100 tags on each strand and experimented on different distances between two adjacent binding sites, from 10 bp to 100 bp. As shown in Figure 3D, the proposed method can successfully identify the two sites when their distance is as close as 40 bp. The further apart the two sites are, the more accurate the detections are. When the distance is 40-60 bp, 75.7% of the detected sites are within 20 bp around the true sites on average; when the distance increases to more than 60 bp, 77.1% of the sites are within 10 bp on average. When distance is less than 20 bp, the method tends to report one binding sites at approximately the center of the two true binding sites. Also, considering the length of binding motifs, it is less likely that the same TF can bind to two motifs too close to each other. Therefore, we set the SeqSite software to combine two identified sites into one if they are closer than 20 bp from each other (see Methods).

### Application to the GABP, STAT1 and NRSF ChIP-seq datasets

**Figure 4:**
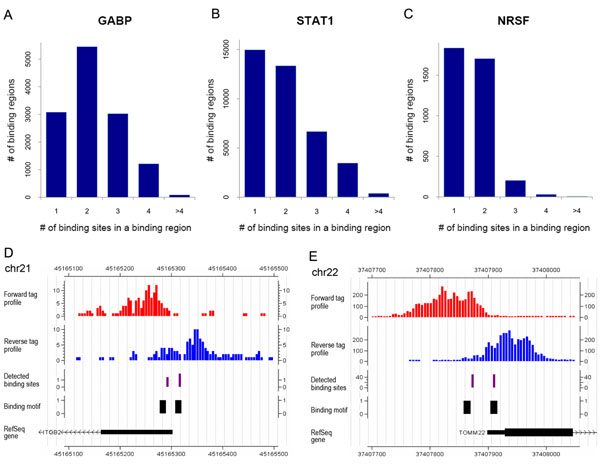
**Binding site identification results by SeqSite on the GABP, STAT1 and NRSF ChIP-seq data.** (A-C) The histograms of the number of detected binding regions with 1, 2, 3, 4 and >4 binding sites for the three datasets, respectively. (D,E) Two examples of the detected multiple binding sites from the GABP data. Both binding regions are in the promoter regions of genes *ITGB2* (also known as *CD18*) (D) and *TOMM22* (E). The tracks from top to bottom are: the forward tag profile, the reverse tag profile, the detected binding sites by SeqSite, binding motifs, and gene annotation.

We applied SeqSite to three ChIP-seq datasets: two of human transcription activators GABP [28] and STAT1 [21] and one of the human transcription repressor NRSF [28]. Table 1 summarizes the detected binding regions at FDR 10% and individual TFBSs. Among all the detected binding regions, SeqSite identified 9,786 (76.0%), 23,914 (61.0%) and 1,948 (51.5%) regions that contain multiple binding sites in the GABP, STAT1 and NRSF data, respectively. The average fragment lengths that were estimated from data are also given in the table. In more details, Figure 4A-C give the histograms of the number of binding regions with 1, 2, 3, 4 and >4 binding sites for the three datasets. A significant portion of the genomic binding regions of these TFs contain more than one binding sites, which explains why the forward tag stacks overlap with the reverse tag stacks. On GABP dataset, we found that most of the binding regions have two or more adjacent binding sites, which is consistent with the literature that GABP binds in a more distributed manner [33]. Several previous publications based on biological experiments have reported that GABP-targeted genes have more than one adjacent binding motifs [39-41], and it has been shown that all the potential binding sites can be functional. For the other two TFs, our results show that the multi-binding events also ubiquitously exist across the genome.

As the true answers of accurate binding sites are not available on real data, we adopted the strategy to use the occurrence of known TF-associated motifs as an indicator of true detection and use the distance between the detected binding sites and motif centers as an indicator for the detection resolution. GABP, STAT1 and NRSF are all well-studied transcription factors. Previous studies on these ChIP-seq data have shown that many detected binding regions are associated known canonical motifs of these factors [6,20,21,28,31]. We extracted the position weight matrixes or PWMs of these binding motifs from the TRANSFAC database [42] and studied their occurrence in the detected binding regions. The sequence LOGOs of the TF-associated motifs are shown in Supplementary Figure S5 in Additional File 1. GABP is associated with a 12-bp canonical motif [28]. STAT1 is associated with three motifs under the data-generation condition: the GAS (gamma-activated sequence) motif and the ISRE motifs (ISRE-3 of 15 bp and ISRE-2 of 14 bp) [20,31]. We took these motifs together to scan for their occurrence in the STAT1 binding regions. NRSF is associated with a 21-bp canonical motif, and its left and right half-site motifs can also be enriched in detected binding regions [6,28,31]. Therefore, we scanned for the occurrence of both the canonical motif and half-site motifs in the detected NRSF binding regions. In the scanning, we used MAST [43] to identify the sequence matches with *P*-value < 0.001.

Two examples of the detected binding regions are given in Figure 4D,E to demonstrate the performance of SeqSite. SeqSite detected two adjacent binding sites at each promoter region of genes *ITGB2* (also known as *CD18*) and *TOMM22*. We can also find two close occurrences of the corresponding sequence motif in each region. It has been reported that the two close GABP binding sites at gene *ITGB2* are both essential for transcriptional activation [39-41]. More examples of the three datasets can be found in Supplementary Figures S6, S7 and S8 in Additional File 1.

### Comparison with other methods on binding site detection accuracy

We compared the performance of SeqSite on binding region/site detection with other existing methods. Three classic methods QuEST [28], MACS [29], SISSRs [31] and two recent methods GPS [37] and PICS [36] were compared. We chose FDR at 10% for MACS and SISSRs, *Q*-value cutoff at 0.01 for GPS, and used the default parameters for other programs (see Methods for details), and applied them to the three ChIP-seq datasets for detecting genome-wide sets of TF binding regions and/or TFBSs. Supplementary Table S1 in Additional File 1 summarizes the results.

We first compared SeqSite with other tools on the accuracy of detected binding sites. Since no true answers are available on the real data, we adopted the motif occurrence as an indicator. Although it must under-estimate positive detections, this evaluation is fair for any particular method. Besides, the detected binding sites and sequence motifs may not necessarily overlap with each other so we extend binding sites to *standardized binding regions* or SBRs for comparison. We did not directly use the binding regions provided by some of the tools compared, because the sizes of those regions were not comparable (Supplementary Figure S9 in Additional File 1). A SBR is a 200-bp genomic region centered at the detected binding site (SeqSite, SISSRs, GPS and PICS) or the binding region summit (QuEST and MACS, as did in literature [34]). We define the *positive detection rate* or PDR as the fraction of SBRs with corresponding motif occurrence.

**Figure 5:**
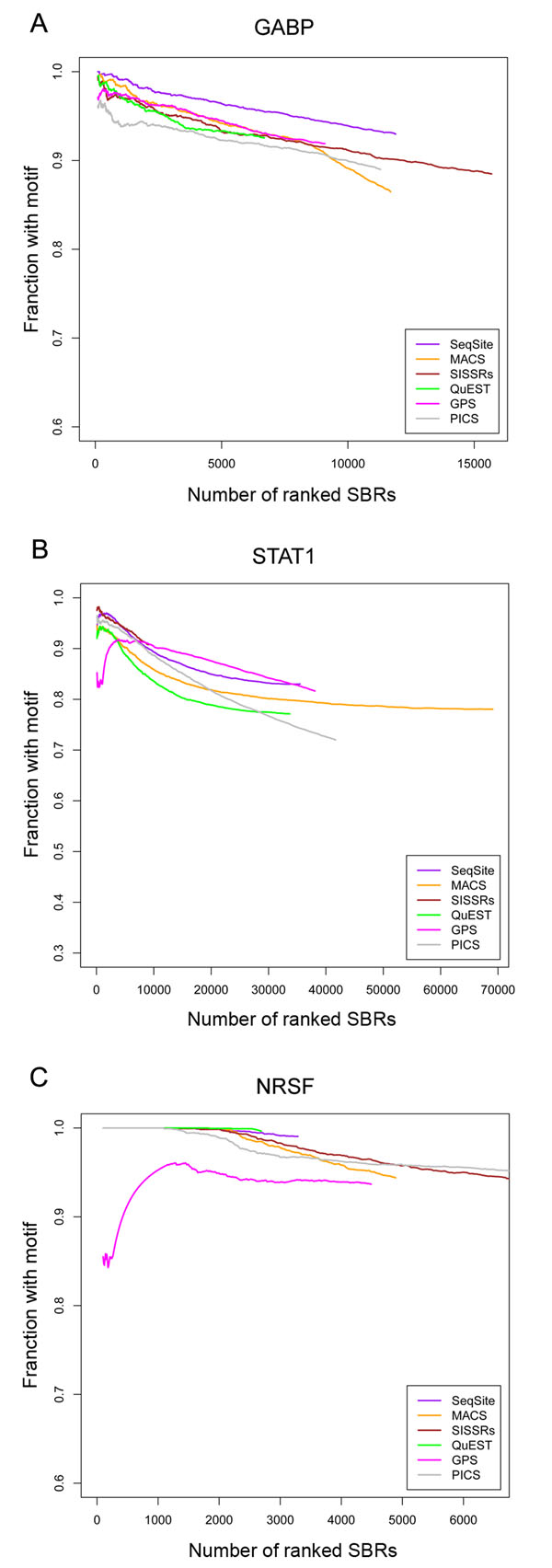
**Positive detection rate (PDR) of binding sites with the six methods.** The positive detection rate is defined as the fraction of SBRs covering associated binding motifs. SBRs are 200-bp standardized regions centered at each binding site detected. The three datasets are the GABP (A), STAT1 (B), and NRSF (C) ChIP-seq datasets. The experimented methods are: SeqSite (purple curve), MACS (orange), SISSRs (brown), QuEST (green), GPS (magenta), and PICS (grey).

The PDRs of each tool compared are shown as a function of the increasing number of ranked SBRs according to their statistical significance (Figure 5). The proposed method SeqSite preformed better than others (GABP) or in the top class among all methods compared (STAT1 and NRSF). On the three datasets, the PDRs of most methods were high (>90% for GABP and NRSF; >80% for STAT1), indicating the canonical motifs of corresponding TFs were highly conserved and most tools performed very well on detection specificity. Except for GPS on STAT1 and NRSF datasets, the PDR curves went down when more SBRs were included, suggesting the significance metric of those methods, including SeqSite, were well defined to rank binding sites detected. Besides, we also investigated the overlapping of SBRs given by different tools (Supplementary Tables S2-S4 in Additional File 1).

### Comparison on binding site detection resolution

The major merit of the proposed SeqSite method is that it can detect binding sites at a high resolution, and can locate closely spaced binding sites. We demonstrated this merit by comparing SeqSite with other tools on detection resolution.

**Figure 6:**
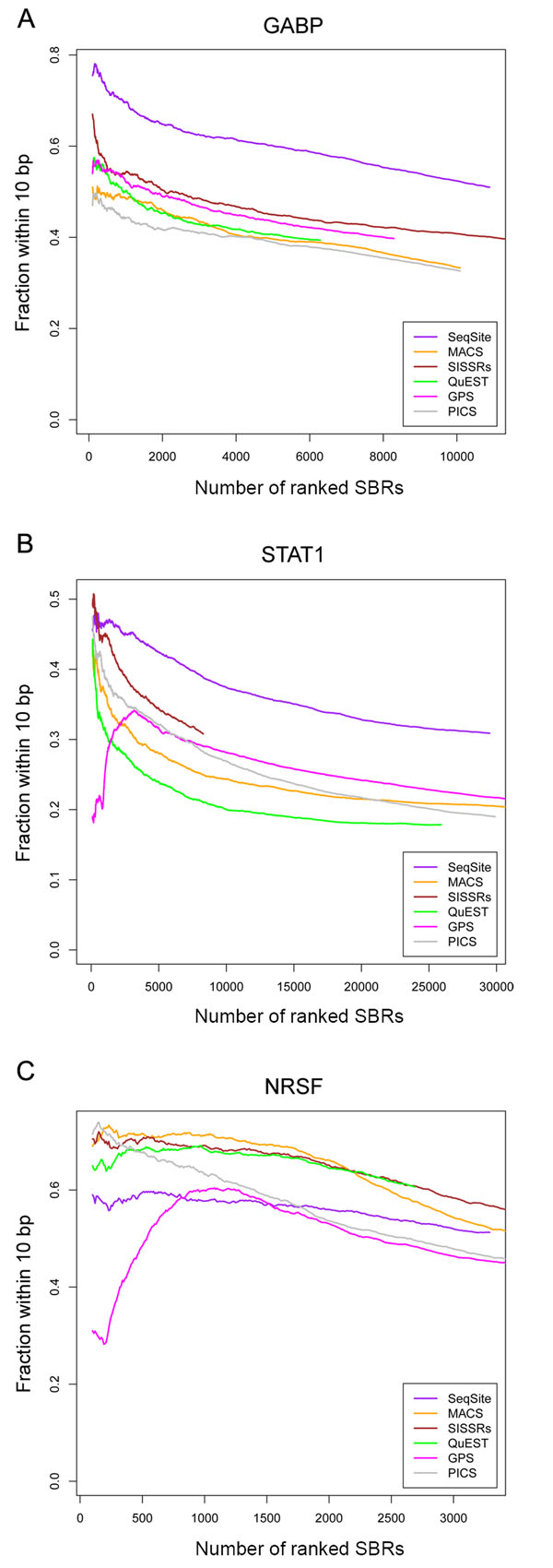
**Resolution of binding sites detected with the six methods.** The binding site identification resolution shown here is indexed by the fraction of SBRs with at least one binding site that is within 10 bp from an associated motif center. The three datasets are the GABP (A), STAT1 (B), and NRSF (C) ChIP-seq datasets. The experimented methods are: SeqSite (purple curve), MACS (orange), SISSRs (brown), QuEST (green), GPS (magenta), and PICS (grey).

To assess the resolution of the method on the real data, we analyzed distances from detected binding sites to their nearest binding motif centers in the true-positive SBRs. Supplementary Figures S10-S12 in Additional File 1 show the histograms of the distances on each dataset. For better comparison, we also measured the fraction of the SBRs with one detected binding site within 10 bp of the nearest motif center. The results are shown in Figure 6. SeqSite outperforms all other methods on the GABP and STAT1 data by a noticeable margin, and the investigated fractions by SeqSite on the GABP data (ranging from 0.51 to 0.78) are higher than those on the STAT1 data (ranging from 0.31 to 0.48). Supplementary Figures S6 and S7 in Additional File 1 give a few examples showing the binding sites detected by SeqSite and other methods. These examples indicate that the other methods usually reported the centers of two adjacent binding sites as their putative TFBSs, and therefore lost some resolution. The pinpointing step based on tag-profile modeling does improve the resolution for detecting both single and multiple binding sites.

On the NRSF data, however, we found that SeqSite’s performance is behind that of MACS, QuEST and SISSRs, although the investigated fractions given by SeqSite (all above 0.51) are higher than those on STAT1. To understand why this happens, we looked into some examples in more details. We found that there are cases where SeqSite gives reasonable multiple binding site detections but doesn’t gain on the resolution measured in this way. For example, in Supplementary Figure S8D in Additional File 1, the long overlap between tag clusters on both strands indicates there must be two adjacent binding sites, which is also cross-validated by the occurrence of the canonical sequence motif. However, because the two motif centers are too close to each other and the canonical motif is relatively long, the two binding sites identified by SeqSite do not result in shorter distances to each motif center, compared with the distance from the single site in the middle to the nearest motif center. We noticed that the recent methods PICS and GPS also perform less well on NRSF data. This reflects that there must be different patterns in NRSF binding that need further investigations comparing to the other TFs.

## Discussion

The interaction of transcription factors and other proteins with genomic DNA is a major knot in the complex system of molecular regulation in cells. Profiling DNA binding sites of these factors at a high resolution is an important step in understanding their functions and regulation networks. It has been reported that some transcription factors have multiple binding sites on DNA at close locations [39-41,44][45][46]. We developed the method SeqSite that can detect such clustered multiple binding sites at a high resolution from ChIP-seq data. Experiments on the GABP, STAT1 and NRSF ChIP-seq data show that a large portion of TF binding events are associated with multiple adjacent binding sites. In recent years, understanding on the function of transcription factors has been widened. They not only play the role of turning on or off their target genes, but also regulate the gene expression in a quantitative manner. Pinpointing the details of their binding on DNA is crucial for better understanding of the mechanism.

Many existing work depends on a secondary step of sequence motif analysis for locating binding sites in detected binding regions. However, due to the complexity of the protein-DNA interaction mechanism such as the involvement of co-factors, sequence motifs are not sufficient for locating binding sites precisely [47-49]. It is also widely observed that many genomic regions with sequence motifs are not bind or not always bind by the corresponding proteins [48,50]. Chromatin-IP followed by next-generation sequencing provides protein-DNA binding tags at a single-base resolution. By modeling the ChIP-seq signal, methods like SeqSite provide a direct way in pinpointing protein binding sites accurately. The detected high-resolution binding sites can also help analyze sequence features of binding sites where no known motif can be found, and therefore identify possible co-factors.

The current version of SeqSite needs to estimate the fragment size in modeling tag profiles from a binding site. This estimation is assumed to be the same for all binding sites in the same experiment. However, there is a randomness in the size of ChIP-ed DNA fragments from different genomic regions with respect to nucleosome position [51]. This partially explains cases where SeqSite may lose resolution and points to a possible direction for further improving the method. Integrating ChIP-seq data with other kinds of data such as nucleosome positioning data would be a promising approach to improve the resolution of binding site pinpointing and to better understand the data. Another possible improvement is utilizing multi-hit reads by some probabilistic methods such as Gibbs sampling [52], or integrating multi-hit reads in the model-fitting procedure in SeqSite.

## Methods

### ChIP-Seq datasets

The three TF ChIP-seq datasets used in this study are: human transcription activator GABP (growth-associated binding protein) in Jurkat T lymphoblast cell [28], transcription activator STAT1 (signal transducers and activator of transcription) in interferon γ-stimulated (IFN-γ) HeLa S3 cell [21], and transcription repressor NRSF (neuron-restrictive silencer factor) in Jurkat T lymphoblast cell with a polyclonal antibody [28]. We downloaded the GABP and NRSF datasets from [53], and the STAT1 data from [54]. The three datasets were all sequenced by the Illumina Genome Analyzer, using single-end sequencing with read length of 25~27 nt. All the datasets provide mapped results of short reads against the human genome (hg18 or NCBI build 36), and total numbers of mapped read-tags are 7,862,231, 26,731,492 and 8,813,398 for GABP, STAT1 and NRSF, respectively. The corresponding control data for GABP and NRSF are of 17,404,922 tags, and the control data for STAT1 contain 23,435,631 tags. Unmapped reads and non-uniquely mapped reads are excluded in this study.

### Detecting tag-enriched regions by tag clustering

Known from the ChIP-seq protocol, tags will be enriched around binding sites of the studied protein. Usually, in large mammalian genomes such as the human genome, there are lots of desert regions with no or few tags, so efficient ChIP-seq analysis algorithms first filter out those regions, only leaving tag-enriched regions for further analysis.

Instead of scanning the genome with a sliding window for tag-enriched region, SeqSite clusters nearby tags within *d*-bp (*d* = 30 by default) to form tag clusters, and the corresponding regions are regarded as tag-enriched regions. This avoids the extra step for merging neighboring enriched regions with a fixed sliding window-size. To remove experimental noise and/or artifacts [26] and to avoid possible false positives in binding site detecting, SeqSite filters clusters with fewer than 10 tags or of less than 100-nt length. We noticed on real data that there are some binding regions with jagged tag profiles, which will poison binding site detection, so SeqSite filters those short tag clusters by setting the cluster length threshold (100-nt).

### Hypothesis testing for tag enrichment

To assess whether a clustered tag-enriched region is caused by random noise, we carry out a hypothesis testing for each region. Researchers originally adopted Poisson distribution to model tag distribution along the genome [13,29], but recent reports indicated that the tags are not uniformly distributed along the genome due to biological and technical biases including local chromatin structure [29], DNA amplification and sequencing bias [55], and sequenced read mappability [21]. Therefore, we use a dynamic parameter *λ*_local_ for the Poisson model, which is modified from MACS [29]. We define *λ*_local_ for each tag cluster without control data as:

where *λ*_BG_ is the background parameter estimated from the whole genome, *λ*_5k_ and *λ*_10k_ are parameters estimated from the 5-kb or 10-kb window centered at the middle position of each tag-enriched region in the ChIP-seq data itself. For each tag cluster with control data, *λ*_local_ is defined as:

where *λ*_control_ is estimated in the control data from the same region where the tags are enriched in the ChIP-seq data, *λ*_control_1k_ is estimated from the 1-kb window centered at its midpoint, and *R*_control2chip_ is the normalization ratio for control against the ChIP-seq sample. As *λ*_local_ also reflects the regional noise level, only the bases with tag density profile above the noise level are regarded as binding region. Thus, we can define detected binding regions’ boundary accordingly.

To calculate *λ*_BG_ , we use the efficient genome size as 80% of the whole human genome when the short read length is ~30 nt, as did in SISSRs [31]. In SeqSite, we set 2.4 G-bp (0.8× 3 G-bp) as the default effective genome size for the human genome. But as the read length changes, the effective genome size should be changed correspondingly [21].

### Correction for multiple testing

We follow Benjamini and Hochberg [56] in adjusting the Poisson *P*-values to correct for occurrence of false positives. All tag clusters which are tested for statistical significance are ranked by *P*-value from most significant to least. Thus, the *Q*-value for each tag cluster is given by:

where *Count* is the total number of tag clusters tested, and *Rank* is each *p*-value’s rank from the smallest to the largest. Tag-enriched regions are then selected by a *Q*-value threshold (the given FDR) instead of a *P*-value threshold.

### The density of tag-enriched regions

For any tag-enriched region, we define its density as the total tag count divided by the length of this region. We sort all tag-enriched regions by density from high to low. A proportion (say 5%) of top-ranking regions are used for estimating the DNA fragment size, which is a parameter of our tag profile model, and also used for investigating the distances in Figure 1. For example, in a mammalian genome, there are about 10,000 binding regions for a TF. By default, we choose the top 5% (~500 regions) with the strongest tag signals to achieve a reliable and robust estimation. Other choices on the parameter of top-percentage were also examined (for fragment size estimation, see *d*_1_ in Figure 1D and Supplementary Figure S2 in Additional File 1).

### DNA fragment size estimation

The estimation of average DNA fragment size is a key step in modeling tag profiles. This is done within the densest tag-enriched regions in the data. For each selected tag-enriched region, the distance between the density centers of the forward tag cluster and the reverse tag cluster is calculated (*d*_1_ in Figure 1C). The average distance is taken as the estimate of average DNA fragment size. If the DNA fragment length mean is known from the experiment protocol, one can set this parameter with this value instead of estimating. But it is recommended to estimate this parameter from data [26].

### Modeling the tag profile

Following previous work on ChIP-chip studies [57,58], we assume that the ChIP-ed DNA fragment size (length) *l* in an experiment follows a gamma distribution *p*(*l*) = *Gamma*(*l|α*,*β*). Based on our observation on the real data, we set the shape parameter *α* = 10. In binding site detection (see below), the largest maximum *R*^2^ value in least-squares fitting is achieved when *α* = 10, comparing with other *α* values (1, 2, 5, 20, 50, and 100) on both the GABP and NRSF ChIP-seq datasets. The other parameter can be written as *αβ*, which is the mean of the gamma distribution, and can be estimated as the average DNA fragment size *L* from data.

Note that each DNA fragment has two arms around a single binding site. We denote *q*(*m*) as the probability that an arm of the DNA fragment is of length *m.* Since the convolution of two identical gamma distributions is a gamma distribution with twice the mean [57], *q*(*m*) is also a gamma distribution but with half the mean of *p*(*l*) (Supplementary Figure S13A in Additional File 1, *L* = 200). By summing over all fragments of at least length *d*, we obtain the fragment coverage function at the position *d* nt from the binding site (Figure S13B in Additional File 1):

where *D* denotes the maximum of *l*, decided by the fragment size selection in the ChIP-seq protocol. We use the sequencing starting positions (the 5’-ends of sequenced DNA fragments) to represent the corresponding tags, and get the tag density as (Figure S13C in Additional File 1):

From Figure S13D in Additional File 1 we can see that for a wide range of *d* <*L* <*D*, we have , and thus we can ignore the multiplicative term . For the simplicity of computation, the tag density at the position *d* nt (*d* <*L*) from the binding site can be approximated by (Figure S13C in Additional File 1):

### Detecting binding sites by least-squares fitting

Given the modeled tag density profile from a binding site, we adopt a least-squares fitting technique to identify TF binding sites. In this case, the least-squares fitting strategy measures the difference between the theoretic tag profile and the observed signal. A sliding window is adopted to scan each tag-enriched region, and sites supporting the least difference are reported as binding sites.

In the least-squares fitting, the *independent variables* are *x_i_* = *f*_tag_ (*i* – 1), *i* = 1,2,…,*W*, where *W* is the width of the sliding window we will use to scan for binding sites. We set *W* as less than the average fragment length *L*, which makes the *d*’s in *f*_tag_ (*d*) approximation better satisfying the restriction that *d* is smaller than *L*. This means that we use only a part of the tag density profile when looking for binding sites.

Meanwhile, we prepare the dependent variable as follows. Given a tag-enriched region, we first count the number of tags starting at each position *n* denoted as *t_n_* (*n* = 1,2,…,*C*; *C* is the size of the tag-enriched region). We then smooth the counts to reduce possible noises introduced in the procedures of fragmentation by sonication and DNA sequencing, and get the smoothed tag density , where *b* is the size of the smoothing window. We set *b* = 20 in our experiments. A proper *b* will reduce noises but not remove adjacent binding information. We use the sliding window with 1-nt step to scan the smoothed tag density *s_n_* ’s along the tag-enriched region. In *j*-th (*j* = 1,2,…,*C-W*+1) sliding window, we take the tag density *s_j_*_+_*_i_*_–1_ as *dependent variables y_ij_* (*i* = 1,2,…,*W*).

In the least-squares fitting, we use the linear function written as:

where *β_j_* is the slope for *j*-th sliding window and *ε_ij_* is a noise term. Based on this model, we compute the slope *β_j_* and goodness-of-fit  for the *j*-th sliding window:

The slope *β_j_* can be used to represent the relative binding affinity. We move the sliding window along the tag-enriched region, and get a goodness-of-fit curve as shown in the example of Figure 2B. A local maximum on this curve indicates a location where the tag density signal best fits the derived model, and is therefore detected as a possible binding site. If there are more than one binding sites in a tag-enriched region, multiple peaks would be observed. We report multiple binding sites from multiple peaks if they are at least 20 bp away from each other. A binding affinity threshold is set according to the signal level of each tag-enriched region to allow only the “strong peaks” detected. In our experiments, we only extract those peaks with regression slope *β_j_* no smaller than half of the maximum slope obtained in each tag-enriched region.

The above procedure is carried out on each DNA strand separately. We take a further step to combine binding sites detected from both strands. Binding sites detected from different strands are reserved unless two sites are too close (less than 20 bp from each other). When two binding sites are close to each other, their tag signal may overlap and shows only one peak on a single strand. But as the signal directions on the two strands are opposite, each strand will detect each of the two sites, and the combination will recover both binding sites. However, considering possible noises and the widths of binding sites, if the locations of the two detected sites from the two strands are within 20 bp from each other, we merge them as one binding sites at the middle. This combination can also rescue some binding sites supported only by one-strand signal because of the exclusion of multiply mapped reads due to mappability.

### Simulation study

We used the derived theoretical tag profile to generate a series of simulated data. For single binding sites, given the position of a binding site (denoted as *p*_bs_ ) and *w* tags in a tag-enriched region for each strand, we randomly placed *w* tags each at a position *p_i_* with the probability *f*_tag_ (*p*_bs_ – *p_i_* ) on each strand. For multiple adjacent binding sites, we generated the tags for each single binding site, and accumulated the tag counts at all positions from all binding sites.

We generated simulation data of different situations: (1) Single binding site, with tag counts varying from 10 to 200 at step-size 10; (2) Two adjacent binding sites of distance 60 bp, with tag counts varying from 10 to 200 at step-size 10; (3) Two adjacent binding sites with tag count 100, and with the distance between the two sides varying from 10 to 100 bp at step-size 10 bp. All datasets were generated in 100 duplications.

### Existing software tools compared

QuEST [28] combines forward tags and reverse tags based on a kernel-density-estimation approach and a “peak-shift” strategy, and then identifies significant peaks and provides peak summits an estimate for TFBSs. MACS [29] models the peak shift size to merge forward and reverse tags for peak detection. The most updated version of MACS can identify sub-peaks and reports the summits of sub-peaks as TFBSs. SISSRs [31] tries to detect the switch points of the forward and the reverse tag profiles and takes the detected points as binding locations. To locate binding sites, PICS [36] adopts a *t*-distribution to model the single-event tag profile [36], while GPS [37] empirically models the tag distribution and refines it during iterations. Another recent method CSDeconv [35] uses a blind deconvolution strategy to locate TFBSs. Because it cannot be applied to large mammalian genomes [35], it isn’t included in the comparison with SeqSite.

We downloaded QuEST-2.4, MACS-1.4.0beta, SISSRs-1.4 and GPS-0.10.1 from their respective websites, PICS release version 1.4.0 from Bioconductor. We chose the FDR for MACS and SISSRs at 10%, *Q*-value cutoff for GPS at 0.01. We applied them on the three ChIP-seq datasets without changing the default setting of other parameters.

### Software availability

We developed a software tool called SeqSite of the method presented. It is written in C/C++ and can run on all major computer platforms with Windows, Unix/Linux. The software tool is available at SeqSite website [38] for free academic use.

## Competing interests

The authors declare that they have no competing interests.

## Authors' contributions

XW developed the method, implemented the software tool, designed the simulation experiments and analyzed the real data. XZ conceived of the study and participated in experiment design, result analysis and discussions. XW and XZ drafted and revised the manuscript. Both authors read and approved the final manuscript.

## Supplementary Material

Additional file 1 **Supplementary Material** Supplementary Material contains all Supplementary Figures and Supplementary Tables. Click here for file
