# Supplementary material for: Pinpointing transcription factor binding sites from ChIP-seq data with SeqSite
Source: BMC Syst Biol. 2011 Dec 14;5(Suppl 2):S3. doi: 10.1186/1752-0509-5-S2-S3 (PMC3287483; doi:10.1186/1752-0509-5-S2-S3)
Supplement: Additional file 1 — Supplementary Material Supplementary Material contains all Supplementary Figures and Supplementary Tables. [file 1752-0509-5-S2-S3-S1.doc]

**Supplementary Material**

**Pinpointing transcription factor binding sites from ChIP-seq data with SeqSite**

**Xi Wang & Xuegong Zhang**

MOE Key Laboratory of Bioinformatics and Bioinformatics Division, TNLIST / Department of Automation, Tsinghua University, Beijing 100084, China


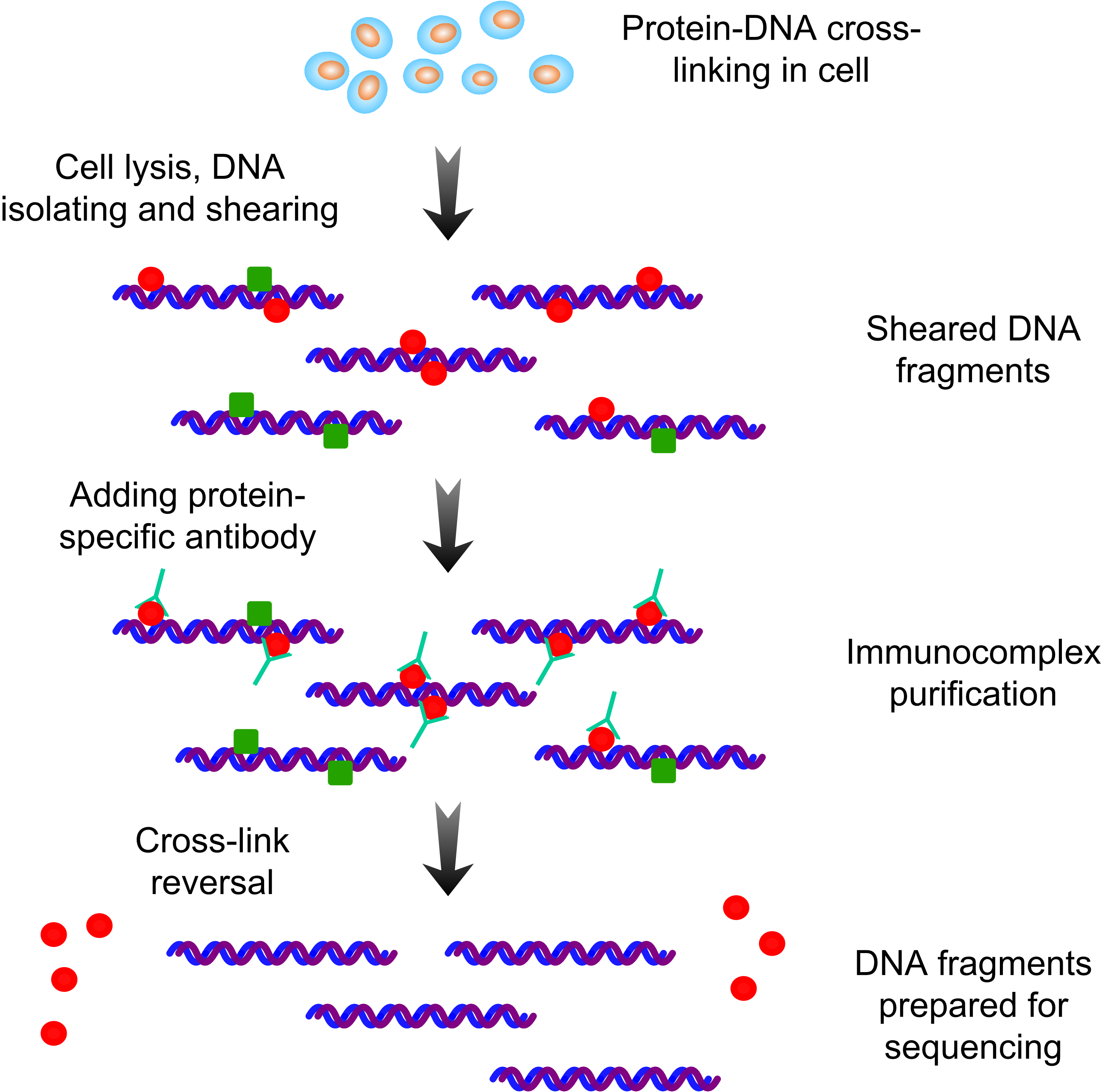


**Figure S1.** The ChIP-seq workflow. The ChIP-ed DNA fragments are then sequenced, and the ChIP-seq data is processed by computational methods to identify genome-wide protein binding regions and/or sites.

(**A**) cluster distance = 20; top percentage = 5


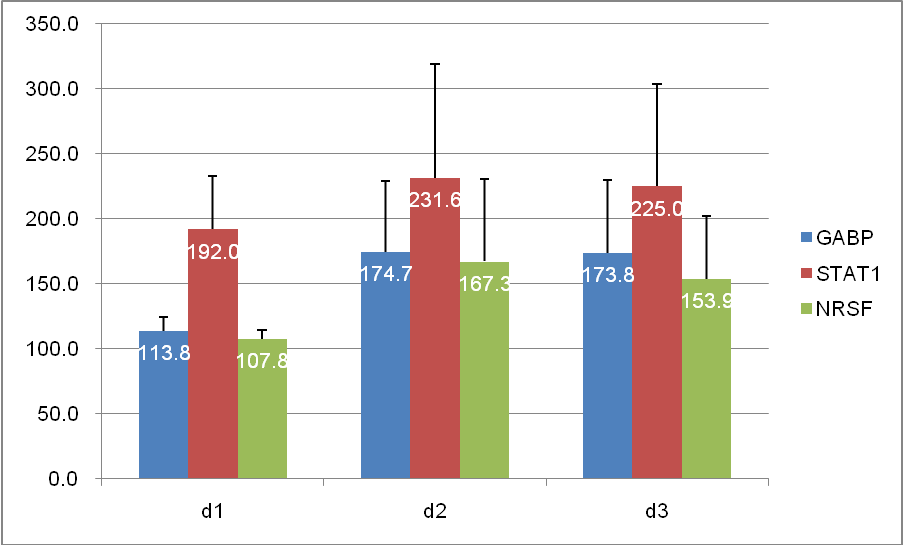


(**B**) cluster distance = 40; top percentage = 5


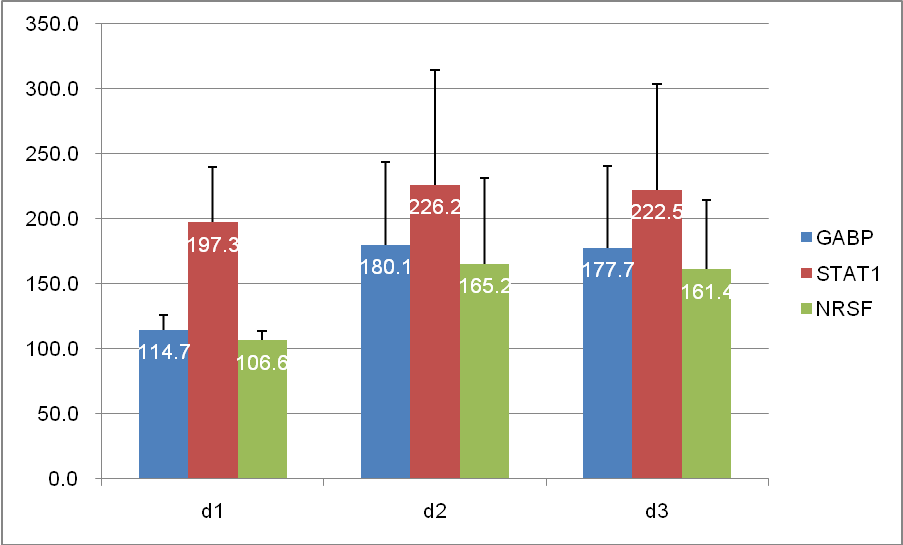


(**C**) cluster distance = 30; top percentage = 2


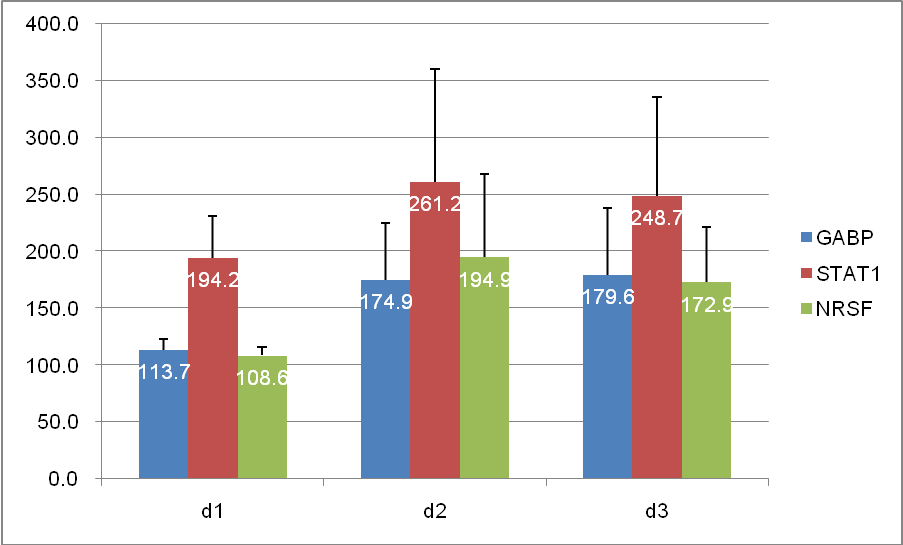


(**D**) cluster distance = 30; top percentage = 10


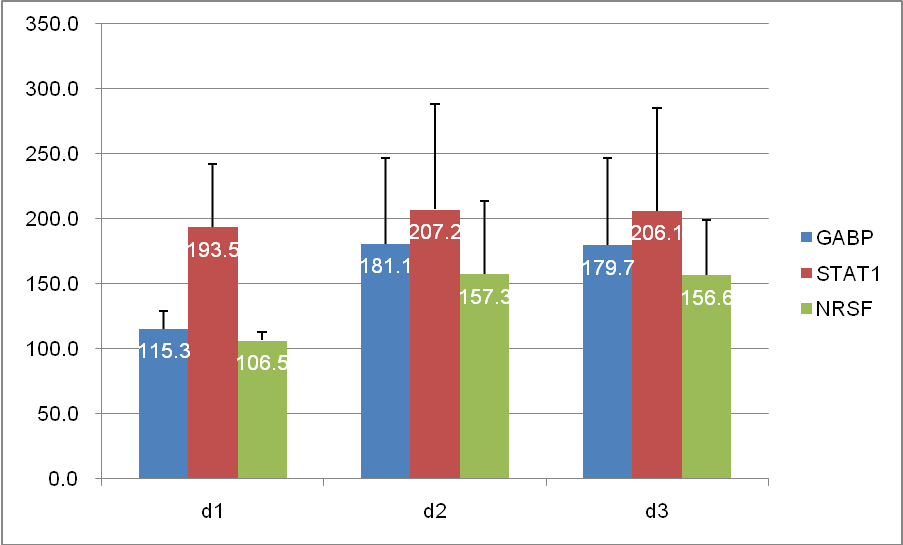


**Figure S2.** Summary of the three distances as shown in Figure 1D in the main text. (A-D) are for different tag clustering distances and different percentages of densest tag clusters investigated.


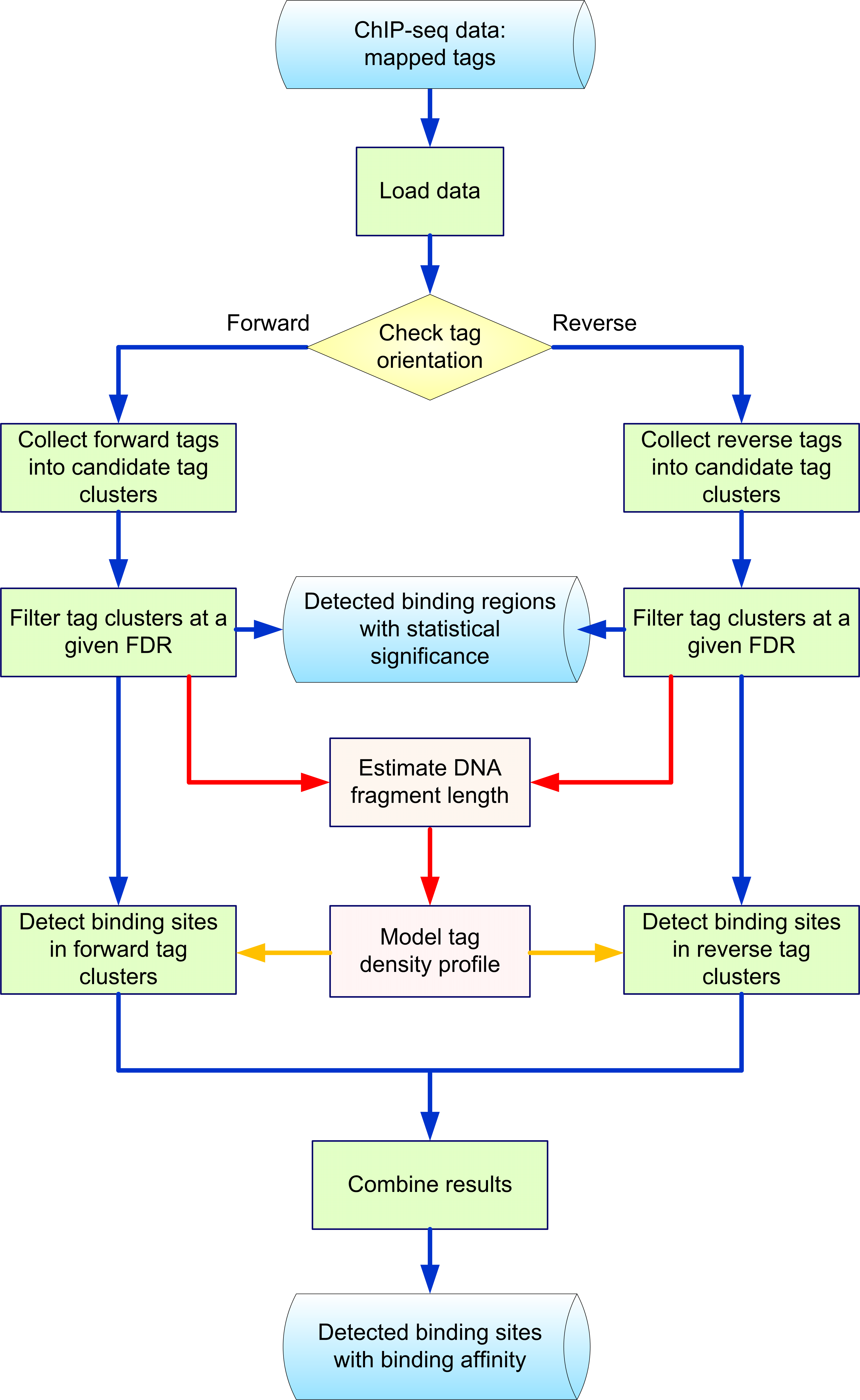


**Figure S3.** The diagram for SeqSite to detect TF binding regions and sites from ChIP-seq data. SeqSite starts with mapped tags, and outputs detected binding regions with statistical significance and detected binding sites with binding affinity.

(**A**) GABP (**B**) STAT1

(**C**) NRSF

**Figure S4.** Distribution of tag counts for each binding region. (A-C) are for transcription factors GABP, STAT1, and NRSF, respectively.

| GABP | |  |
| --- | --- | --- |
| STAT1 | GAS |  |
|  | ISRE-2 |  |
|  | ISRE-3 |  |
| NRSF | Full site |  |
|  | Left half-site |  |
|  | Right half-site |  |

**Figure S5.** Sequence LOGOs for the TF binding motifs. These LOGOs were drawn by enoLOGOS (http://www.benoslab.pitt.edu/cgi-bin/enologos/enologos.cgi).

| (**A**) chr16:30952000-30952500  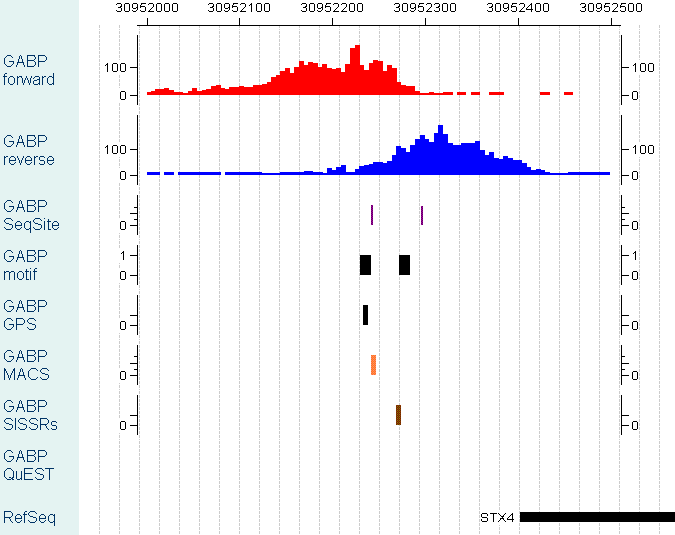 | (**B**) chr22:18259450-18259800  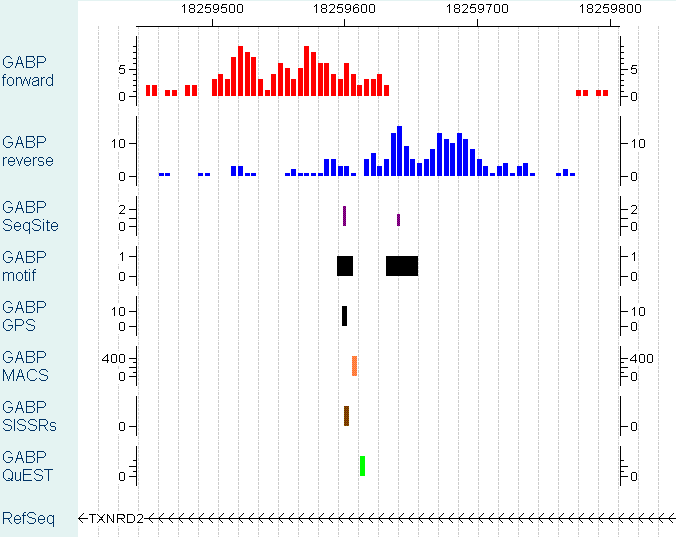 |
| --- | --- |
| (**C**) chr22:18309000-18309600  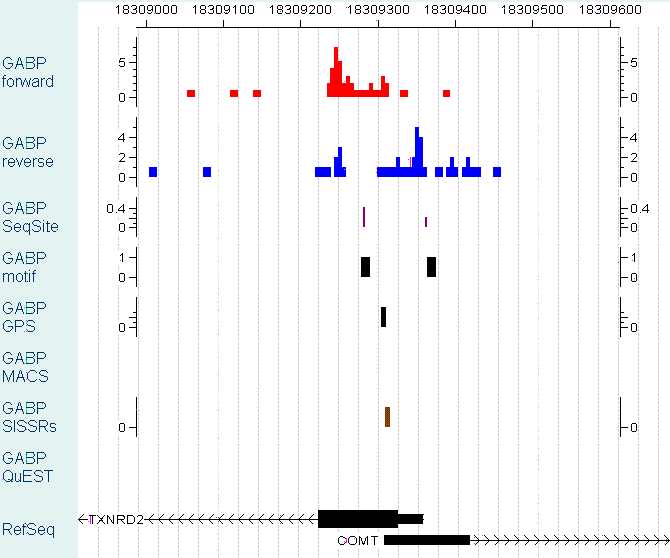 | (**D**) chr22:40408300-40408600  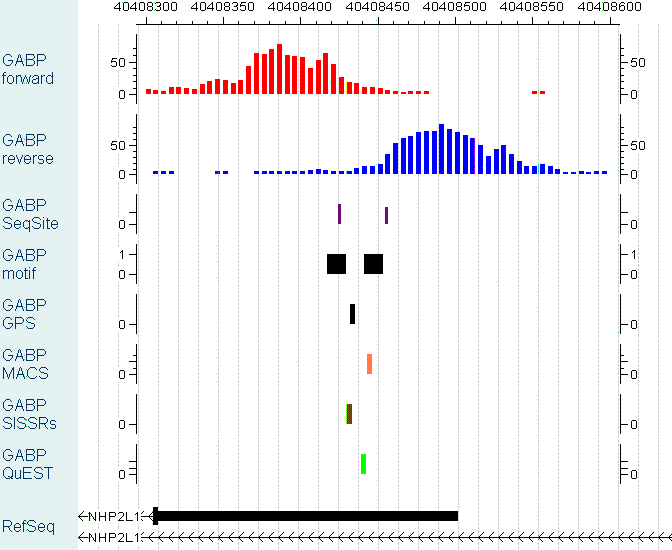 |
| (**E**) chr22:28114400-28114800  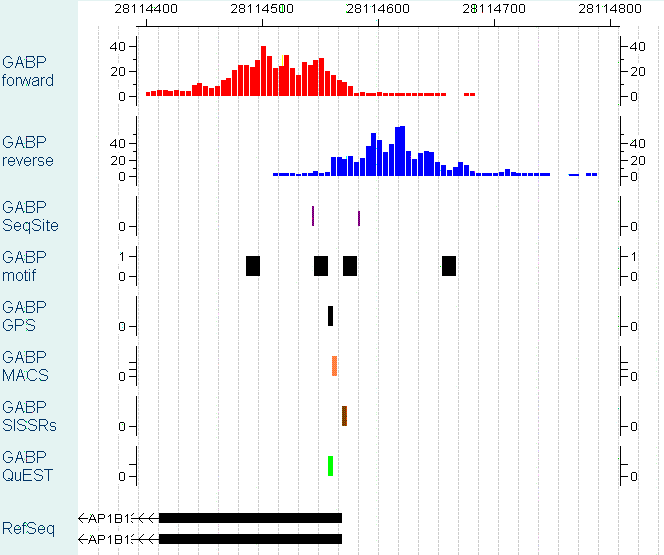 | (**F**) chr22:29082700-29083200  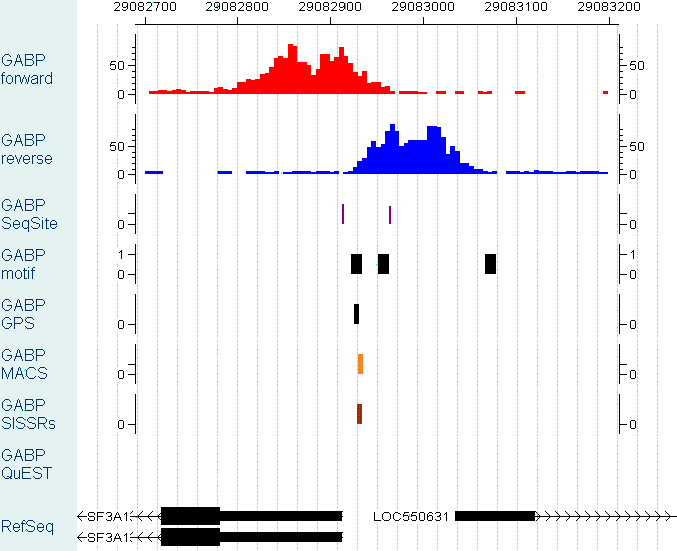 |

**Figure S6.** Examples of the detected binding sites by SeqSite and other methods on the GABP ChIP-seq data. In each panel, the first two tracks are for forward and reverse tag profiles, respectively; the third track shows the detected binding sites by SeqSite; the fourth track shows the associated binding motifs; the next four tracks provide the detected binding sites by GPS, MACS, SISSRs, and QuEST, respectively; and the last track is for RefSeq gene annotation. All the figures were displayed by CisGenome Browser.

| (**A**) chr5:119502700-119503100  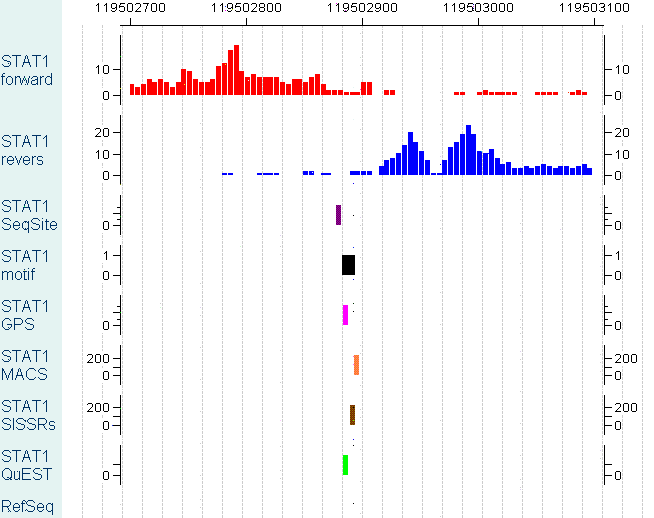 | (**B**) chr21:28630500-28630950  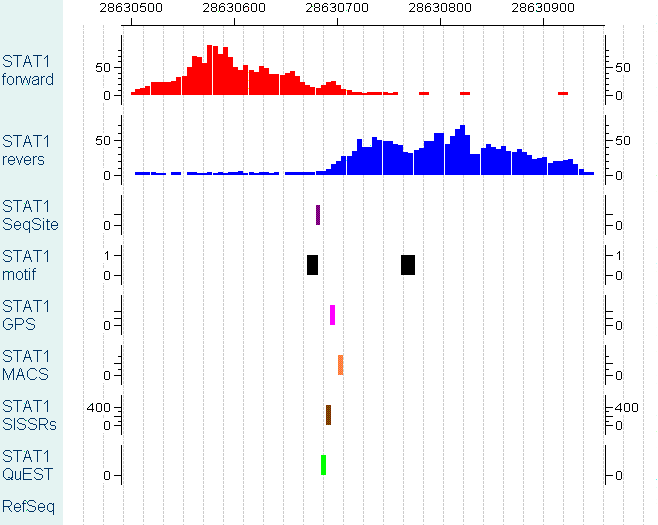 |
| --- | --- |
| (**C**) chr4:167153100-167153600  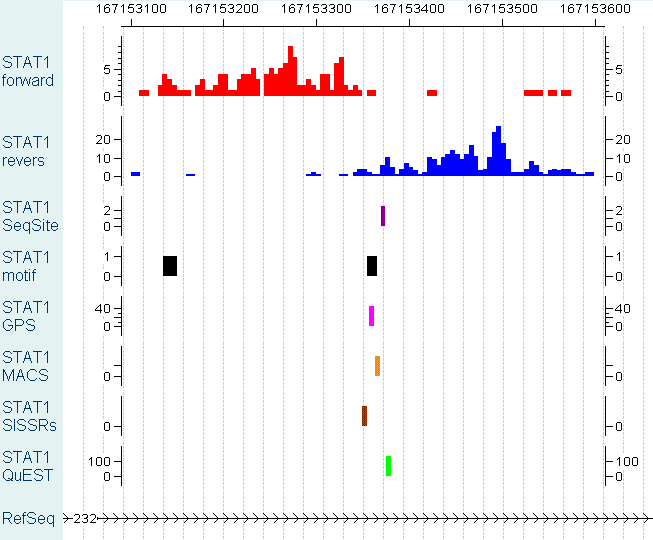 | (**D**) chr14:63400800-63401200  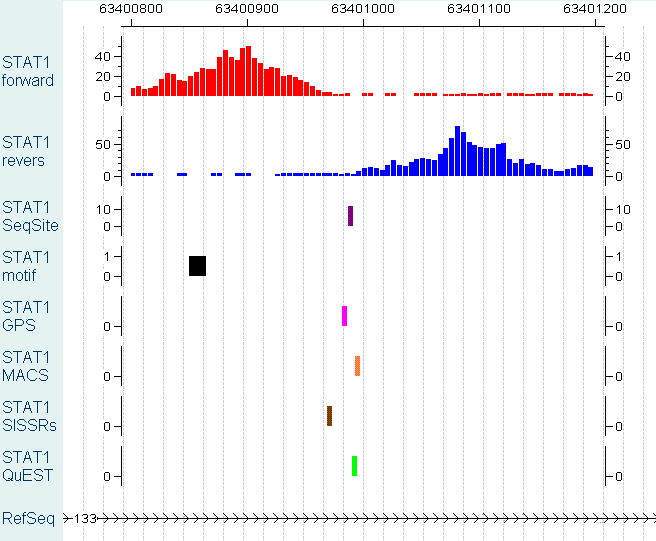 |
| (**E**) chr11:80008900-80009600  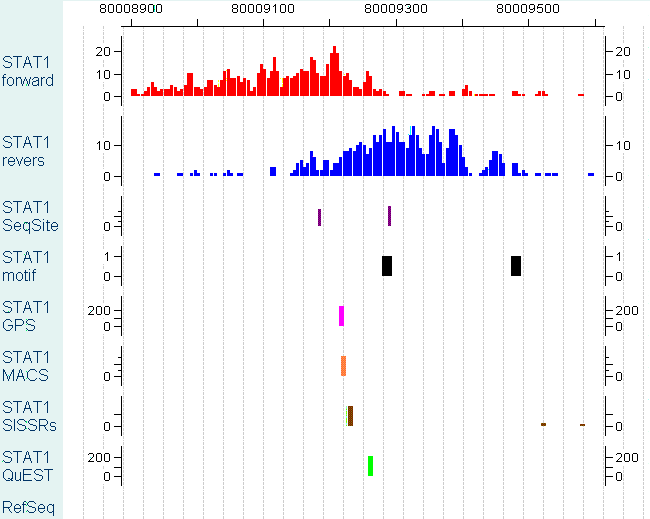 | (**F**) chr12:113721600-113722200  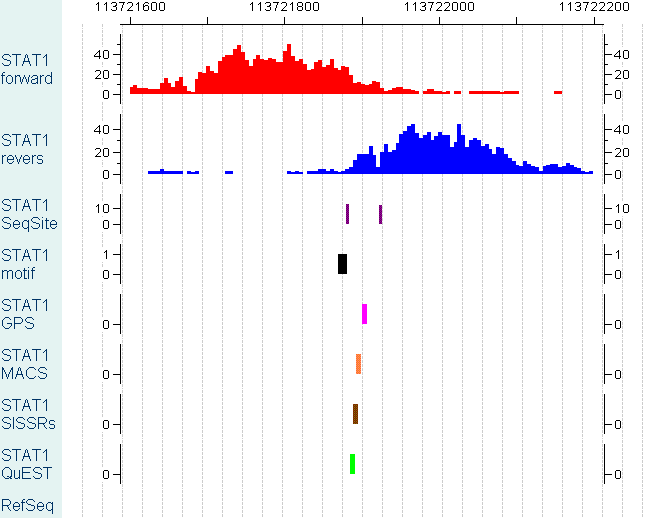 |

**Figure S7.** Examples of the detected binding sites by SeqSite and other methods on the STAT1 ChIP-seq data. In each panel, the first two tracks are for forward and reverse tag profiles, respectively; the third track shows the detected binding sites by SeqSite; the fourth track shows the associated binding motifs; the next four tracks provide the detected binding sites by GPS, MACS, SISSRs, and QuEST, respectively; and the last track is for RefSeq gene annotation. All the figures were displayed by CisGenome Browser.

| (**A**) chr17:37585600-37586100  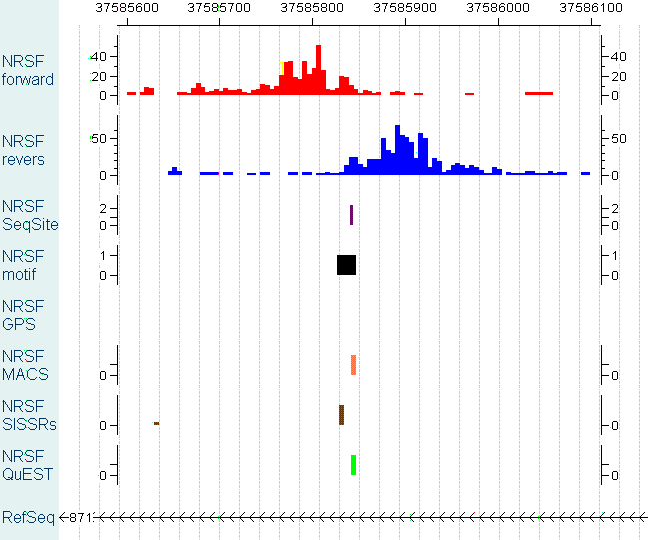 | (**B**) chr1:50506350-50506650  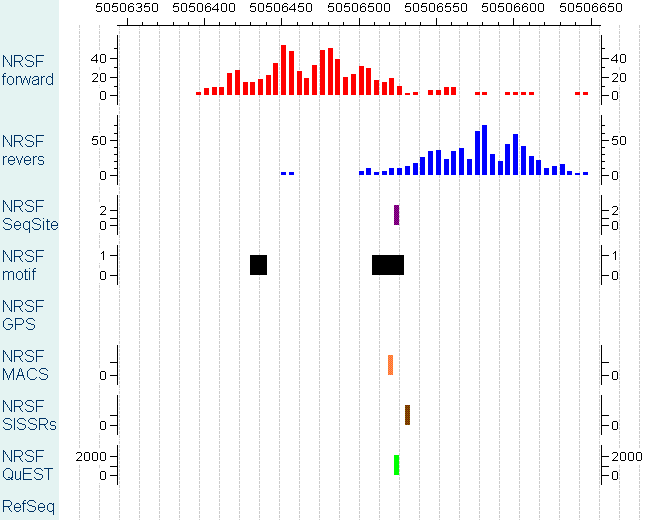 |
| --- | --- |
| (**C**) chrX:74067750-74068150  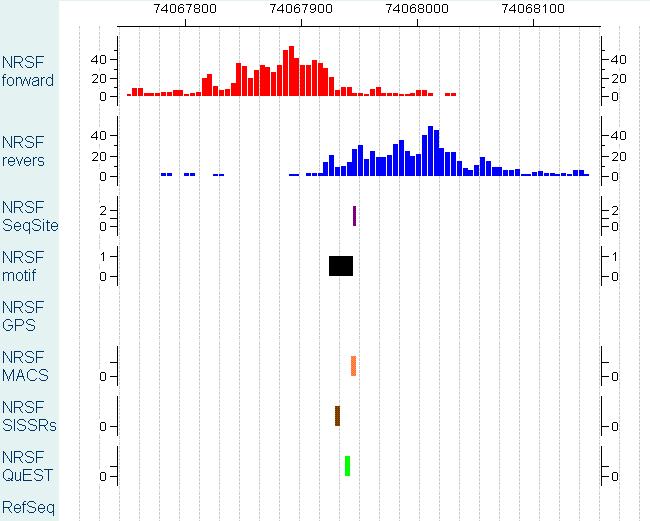 | (**D**) chr8:145531050-145531350  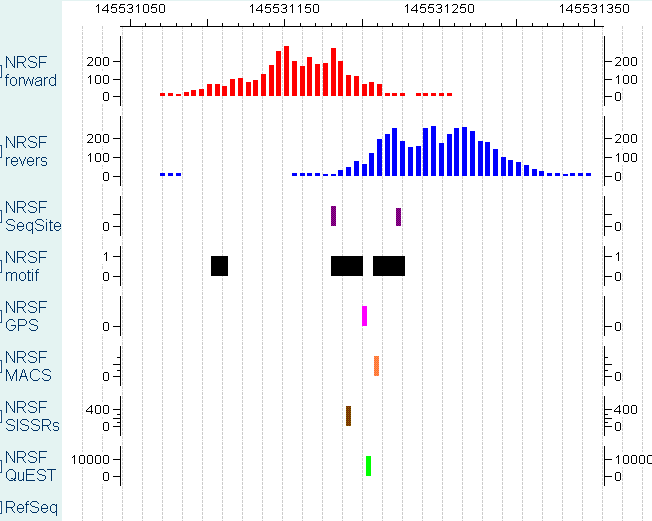 |
| (**E**) chr18:43040700-43041100  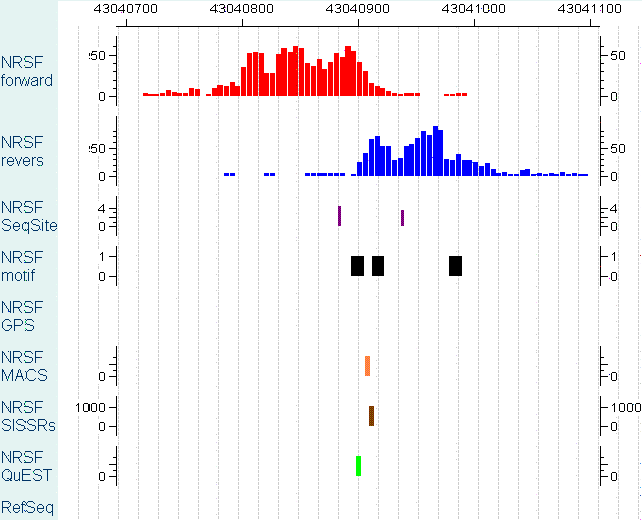 | (**F**) chr14:76744800-76745200  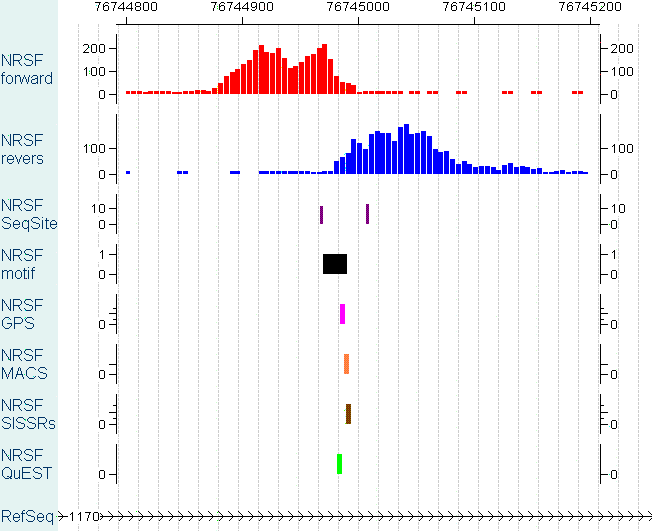 |

**Figure S8.** Examples of the detected binding sites by SeqSite and other methods on the NRSF ChIP-seq data. In each panel, the first two tracks are for forward and reverse tag profiles, respectively; the third track shows the detected binding sites by SeqSite; the fourth track shows the associated binding motifs; the next four tracks provide the detected binding sites by GPS, MACS, SISSRs, and QuEST, respectively; and the last track is for RefSeq gene annotation. All the figures were displayed by CisGenome Browser.

(**C**)

(**D**)

(**A**)

(**B**)

**Figure S9.** Positive detection rate (PDR) of binding regions. (A,C,E) Shown are the fraction of detected binding regions with associated binding motifs; (B,D,F) Shown are box-plots of the lengths of detected binding regions by different methods. As the length of the detected binding regions by different methods varies a lot, this comparison is not so fare for the methods give short regions. However, we can still have an idea that SeqSite offers narrower binding regions but similar or not so worse prediction accuracy. SISSRs provides the shortest binding regions, but the regions may not even cover the associated motifs. GPS and PICS were not included in this comparison because they didn’t report detected binding regions.

(**E**)

(**F**)

SeqSite

MACS

GPS

QuEST

SISSRs

PICS

**Figure S10.** Binding site identification resolution on the GABP ChIP-seq data by SeqSite, MACS, SISSRs, QuEST, GPS, and PICS. In each panel, shown is the histogram of the distances from detected binding sites to their nearest binding motif centers. SeqSite achieves the smallest standard-deviation 24.8.

SeqSite

MACS

GPS

QuEST

SISSRs

PICS

**Figure S11.** Binding site identification resolution on the STAT1 ChIP-seq data by SeqSite, MACS, SISSRs, QuEST, GPS, and PICS. In each panel, shown is the histogram of the distances from detected binding sites to their nearest binding motif centers. In STAT1 data, SISSRs only identified ~10,000 binding regions while other methods ~30,000, so the narrow distribution for SISSRs is mainly due to this lack of binding region calling. However, SeqSite achieves the second smallest standard-deviation 39.5.

SISSRs

QuEST

GPS

PICS

SeqSite

MACS

**Figure S12.** Binding site identification resolution on the NRSF ChIP-seq data by SeqSite, MACS, SISSRs, QuEST, GPS, and PICS. In each panel, shown is the histogram of the distances from detected binding sites to their nearest binding motif centers. SeqSite achieves the second smallest standard-deviation 20.3, but it detects more binding regions than QuEST.

**Figure S13. Plots that help illustrate the derivation of the tag density profile.** (A) The length distribution for ChIP-DNA fragments and fragments’ “arm”. The means for the two distributions are 200 and 100, respectively. (B) The fragment intensity distribution. (C) The tag density distribution. The solid line indicates the full equation, while the dashed line indicates the approximation. (D) The sum term in the approximation.

**Table S1**. Detection results for different methods on GABP, STAT1, and NRSF ChIP-seq datasets.

|  | **GABP** | | **STAT1** | | **NRSF** | |
| --- | --- | --- | --- | --- | --- | --- |
|  | **# BR** | **# BS** | **# BR** | **# BS** | **# BR** | **# BS** |
| SeqSite | 12,875 | 28,846 | 39,222 | 82,104 | 3,786 | 6,047 |
| MACS ^(a)^ | 6,588 | 12,560 | 28,573 | 70,827 | 2,154 | 5,570 |
| SISSRs | 17,245 | 17,737 | 9,514 | 10,645 | 29,176 | 30,739 |
| QuEST | 6,994 | 6,994 | 33,903 | 33,903 | 2,964 | 2,964 |
| GPS ^(b)^ | - | 9,822 | - | 38,368 | - | 4,708 |
| PICS ^(c)^ | - | 11,626 | - | 41,907 | - | 7,617 |

BR: binding regions; BS: binding sites; (a) The summits of MACS-detected binding regions are regarded as binding sites, and the same for QuEST; (b) GPS doesn’t report binding regions; (c) PICS reports binding positions with each a confidence interval, and we treat the mid-point of a confidence interval as the detected binding site.

The false discovery rate (FDR) for SeqSite, MACS, and SISSRs is specified to be 10%; the *Q*-value cutoff for GPS is set to 0.01; FDR cannot be specified beforehand for QuEST, but it reports that the FDR is 1.34% and 1.58% on the GABP dataset for called peaks and detected binding regions, respectively; 4.62% and 5.45% on the NRSF dataset for called peaks and detected binding regions, respectively; on the STAT1 dataset FDR is not available due to the not enough tags in the control data. We ran PICS with its default parameters following the manual and the FDR cannot be specified.

**Table S2**. SBR overlapping summary between every two methods on the GABP dataset.

|  | **SeqSite** | | **MACS** | | **SISSRs** | | **QuEST** | | **GPS** | | **PICS** | |
| --- | --- | --- | --- | --- | --- | --- | --- | --- | --- | --- | --- | --- |
|  | **#** | **%** | **#** | **%** | **#** | **%** | **#** | **%** | **#** | **%** | **#** | **%** |
| **SeqSite** | 12012 | 100.0 | 7450 | 62.0 | 10024 | 83.4 | 6476 | 53.9 | 7598 | 63.3 | 8733 | 72.7 |
| **MACS** | 8043 | 67.5 | 11912 | 100.0 | 8588 | 72.1 | 4975 | 41.8 | 5713 | 48.0 | 6261 | 52.6 |
| **SISSRs** | 10506 | 66.2 | 8469 | 53.4 | 15863 | 100.0 | 6792 | 42.8 | 8469 | 53.4 | 8507 | 53.6 |
| **QuEST** | 6472 | 92.5 | 4932 | 70.5 | 6732 | 96.3 | 6994 | 100.0 | 5692 | 81.4 | 5504 | 78.7 |
| **GPS** | 7624 | 81.7 | 5690 | 61.0 | 8437 | 90.4 | 5695 | 61.0 | 9332 | 100.0 | 6233 | 66.8 |
| **PICS** | 9187 | 79.7 | 6226 | 54.0 | 8573 | 74.4 | 5517 | 47.9 | 6247 | 54.2 | 11526 | 100.0 |

**Table S3**. SBR overlapping summary between every two methods on the STAT1 dataset.

|  | | **SeqSite** | | | **MACS** | | | | **SISSRs** | | | **QuEST** | | | **GPS** | | | | **PICS** | | | |
| --- | --- | --- | --- | --- | --- | --- | --- | --- | --- | --- | --- | --- | --- | --- | --- | --- | --- | --- | --- | --- | --- | --- |
|  | | **#** | **%** | | **#** | | **%** | | **#** | **%** | | **#** | **%** | | **#** | | **%** | | **#** | | **%** | |
| **SeqSite** | 35730 | | 100.0 | 19084 | | 53.4 | | 6157 | | 17.2 | 19374 | | | 54.2 | | 19995 | | 56.0 | | 21810 | | 61.0 |
| **MACS** | 19172 | | 27.7 | 69301 | | 100.0 | | 8010 | | 11.6 | 19583 | | | 28.3 | | 27090 | | 39.1 | | 19185 | | 27.7 |
| **SISSRs** | 6215 | | 66.9 | 7993 | | 86.0 | | 9292 | | 100.0 | 6543 | | | 70.4 | | 8058 | | 86.7 | | 5455 | | 58.7 |
| **QuEST** | 19267 | | 56.9 | 19501 | | 57.5 | | 6519 | | 19.2 | 33886 | | | 100.0 | | 20542 | | 60.6 | | 20250 | | 59.8 |
| **GPS** | 19835 | | 51.7 | 26941 | | 70.2 | | 8034 | | 20.9 | 20545 | | | 53.6 | | 38364 | | 100.0 | | 18801 | | 49.0 |
| **PICS** | 22079 | | 52.7 | 19217 | | 45.9 | | 5459 | | 13.0 | 20329 | | | 48.5 | | 18901 | | 45.1 | | 41896 | | 100.0 |

**Table S4**. SBR overlapping summary between every two methods on the NRSF dataset.

|  | | **SeqSite** | | | | **MACS** | | | | **SISSRs** | | | **QuEST** | | | **GPS** | | | | **PICS** | | | |
| --- | --- | --- | --- | --- | --- | --- | --- | --- | --- | --- | --- | --- | --- | --- | --- | --- | --- | --- | --- | --- | --- | --- | --- |
|  | | **#** | | **%** | | **#** | | **%** | | **#** | **%** | | **#** | **%** | | **#** | | **%** | | **#** | | **%** | |
| **SeqSite** | 3576 | | 100.0 | | 2437 | | 68.1 | | 2996 | | | 83.8 | 2438 | | 68.2 | | 1330 | | 37.2 | | 2000 | | 55.9 |
| **MACS** | 2537 | | 50.1 | | 5061 | | 100.0 | | 4038 | | | 79.8 | 2125 | | 42.0 | | 1150 | | 22.7 | | 1639 | | 32.4 |
| **SISSRs** | 3116 | | 10.9 | | 3920 | | 13.7 | | 28612 | | | 100.0 | 2854 | | 10.0 | | 4013 | | 14.0 | | 3230 | | 11.3 |
| **QuEST** | 2438 | | 82.3 | | 2118 | | 71.5 | | 2828 | | | 95.4 | 2964 | | 100.0 | | 1509 | | 50.9 | | 1979 | | 66.8 |
| **GPS** | 1331 | | 28.6 | | 1149 | | 24.7 | | 4003 | | | 86.0 | 1509 | | 32.4 | | 4656 | | 100.0 | | 1597 | | 34.3 |
| **PICS** | 2021 | | 26.6 | | 1624 | | 21.3 | | 3226 | | | 42.4 | 1979 | | 26.0 | | 1597 | | 21.0 | | 7612 | | 100.0 |

Each count (#) represents the number of SBRs detected by tool A (indicated in each row) and overlapped with at least one SBR detected by tool B (column), and each percentage (%) is the fraction of the overlapped SBRs among all the SBRs detected by tool A (row).

Please note that the number of SBRs detected by tool A overlapping with those by tool B may not be the same number of SBRs detected by tool B overlapping with those by tool A. This is because sometimes a SBR detected by tool A may overlap with two SBRs detected by tool B and vice versa. So Supplementary Tables S2-4 are not symmetric matrices. From the three tables, taking into account the total SBR numbers, the overlapping counts indicate that SeqSite is at least comparable with other tools in terms of consistency and false positive detections.
